# Malaria in Appalachia? A Rare Case of Plasmodium vivax in an Adolescent Exchange Student From South Korea

**DOI:** 10.7759/cureus.69462

**Published:** 2024-09-15

**Authors:** Cora E Miracle, Lauren Tufts, Jacob T Kilgore

**Affiliations:** 1 Pediatrics, Marshall University Joan C. Edwards School of Medicine, Huntington, USA; 2 Pediatric Hospital Medicine, Cabell Huntington Hospital, Huntington, USA; 3 Pediatric Infectious Diseases, Cabell Huntington Hospital, Huntington, USA

**Keywords:** fever, hepatosplenomegaly, jaundice, malaria, thrombocytopenia, traveler

## Abstract

Cultural globalization, including the resumption of international travel post-SARS-CoV-2 pandemic, emphasizes the importance of regional infectious disease variation, especially when considering a differential diagnosis for fever in a traveler. Prompt diagnosis is often imperative to initiate proper treatment and reduce morbidity and mortality associated with many environmental and vector-borne pathogens. The *Anopheles *mosquito transmits malaria in areas endemic to malaria. Malaria, while not endemic to the United States, can be seen in a traveler. This illness can be deadly if left untreated. Symptoms of malaria include but are not limited to jaundice, cyclic fever, and flu-like illness. In this case report, we describe a unique presentation of *Plasmodium vivax* malaria in a 17-year-old traveler from South Korea with a negative rapid malaria test. A peripheral smear from microscopy demonstrated the presence of gametocytes, which are pathognomonic for malaria. Despite the presence of a very low parasitemia (<1%), the patient was noted to have some severe features such as significant thrombocytopenia, acute kidney injury, as well as relapsed disease several months later despite adequate treatment. A high clinical index of suspicion and a detailed history allowed prompt treatment and no permanent sequelae.

## Introduction

Malaria is a parasitic infection transmitted by infected female *Anopheles* mosquitos [1]. While malaria is very well known worldwide, it is not endemic to the United States with only 2,000 cases occurring each year [2]. Therefore, most cases of malaria in the United States are associated with individuals traveling to a country with malaria endemicity. Malaria generally presents with flu-like symptoms, including chills, headaches, muscle aches, and fever [3,4]. The fever pattern of malaria is typically characteristic of this disease and occurs in a quotidian, tertian, or quartan-like pattern [3]. Symptoms of malaria manifest after what is known as the “incubation period” [2]. According to the Centers for Disease Control and Prevention (CDC), an incubation period for malaria can last between 7 and 30 days, easily allowing a patient to finalize travel and return to their respective destination [2]. While malaria and malaria treatment are thoroughly characterized in the literature, its rarity in the United States often leads to misdiagnosis, causing serious morbidity and progression to complicated disease. In this case report, we describe a case of recurrent fever secondary to *Plasmodium vivax* in a young patient traveling from South Korea. This case is unique in that infection within the acquired region of South Korea is relatively rare, and an outbreak of malaria was noted around the time of patient visitation. This patient also experienced severe symptoms such as thrombocytopenia, transaminitis, and relapsed disease despite a low parasite load on presentation.

## Case presentation

**Figure 1:**
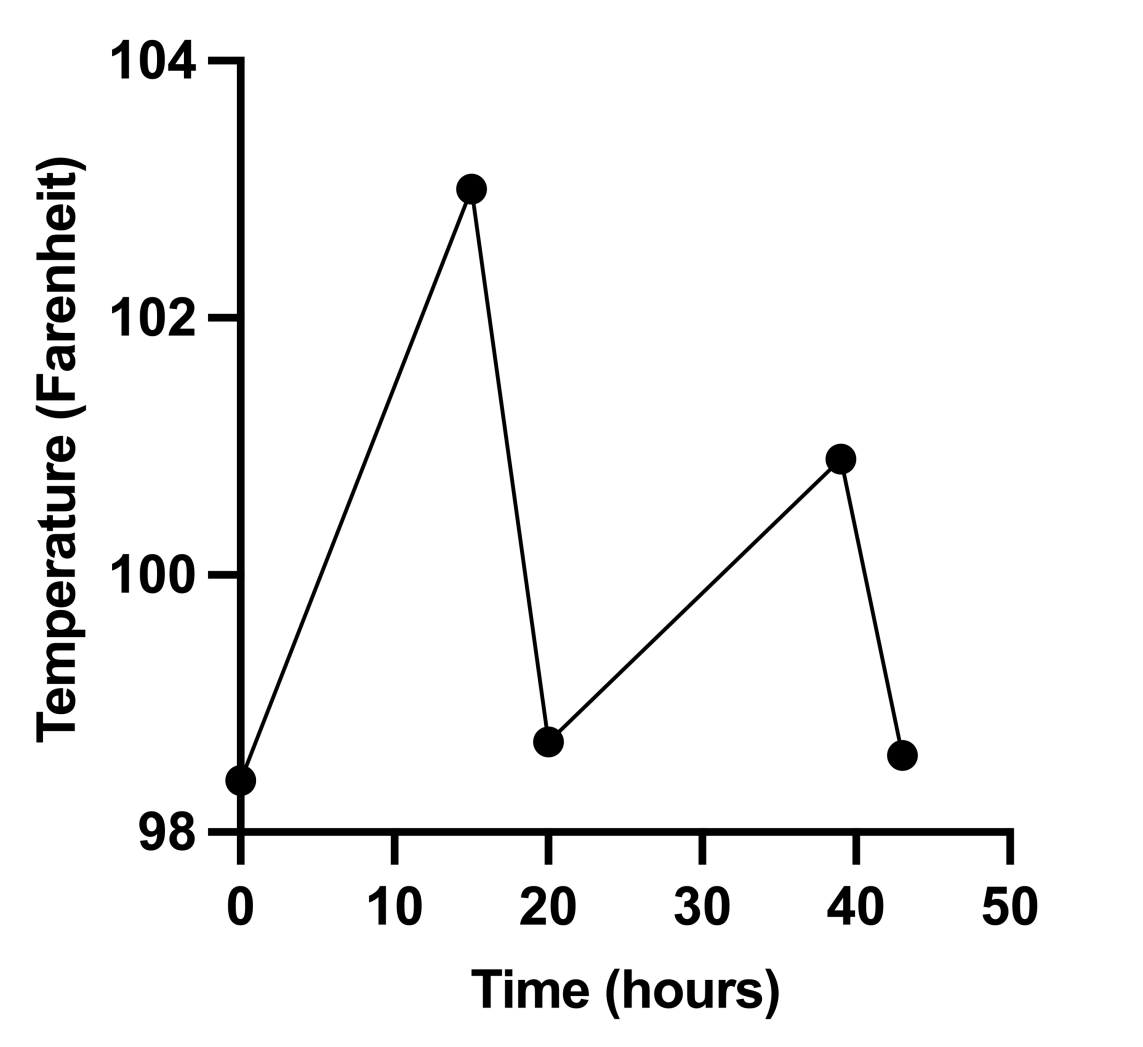
Tertian fever Graph depicting the patient's tertian fever over a 48-hour period.

A 17-year-old South Korean male with no known past medical history was referred to the emergency department (ED) by his primary care physician (PCP) for recrudescent fever and myalgias of two weeks duration. Fevers were accompanied by bloody diarrhea, non-bloody and non-bilious emesis, headaches, and, most recently, scleral icterus. On initial ED evaluation, the patient was afebrile and hemodynamically stable on room air. Sequent temperatures recorded during admission included cyclic fevers (Tmax, 103.1 F; oral) approximately every 48-72 hours (Figure 1). Physical examination was notable for a pale, ill-appearing adolescent with scleral icterus, jaundice, right upper quadrant tenderness, and hepatosplenomegaly. The cardiac exam was benign, with normal rate and rhythm and no murmurs. Lungs were clear to auscultation. Urine was dark in color with a pH of 5.5, but the urinalysis was negative for blood, urobilinogen, bilirubin, glucose, ketones, protein, nitrites, and leukocyte esterase. The urine culture demonstrated no bacterial growth. Fecal occult blood testing was also negative. Further labwork demonstrated normocytic anemia, transaminitis, thrombocytopenia, and hyperbilirubinemia (Table 1). Coagulation factors (PT/PTT/INR) were within normal limits. C-reactive protein (CRP) and procalcitonin were elevated. Acute infectious hepatitis testing was negative, including hepatitis A IgM antibody (HepA IgM), hepatitis B surface antigen (HBsAg), hepatitis B core IgM antibody (HepB Core IgM), and hepatitis C IgG antibody (HepC Ab). A stool PCR pathogen panel including *Salmonella*, *Shigella*, and *Campylobacter* species, among other pathogens, was negative as was a respiratory pathogen PCR panel. An abdominal ultrasound demonstrated hepatomegaly and splenomegaly with no biliary duct dilation and patent hepatic blood flow. A blood culture was obtained, and the patient was started on empiric ceftriaxone (2 gm every 12 hours) and doxycycline (100 mg BID) for potentially implicated bacterial pathogens associated with regional travel, such as Salmonella or tick-borne illness. The Pediatric Infectious Diseases (Peds ID) team was consulted for further management recommendations.

**Table 1:** Laboratory values Relevant laboratory values obtained from patients’ first hospitalization.

Lab	Obtained value	Parameter	Reference Value
Hemoglobin	9.7	g/dL	13-18
Hematocrit	30.4%	-	40-52
Reticulocyte	7.96%	-	0.73-3.12
AST	125	IU/L	10-60
ALT	227	IU/L	7-52
Platelets	70,000	k/cm^2^	150-440
Total bilirubin	2.4	mg/dL	0.3-1.0
PT	11.09	seconds	9.7-12.30
INR	1.01	-	0.9-1.30
PTT	28.56	seconds	21-33
CRP	3	mg/dL	0.0-0.5
Procalcitonin	2.47	ng/mL	0.05-2.0

Using an electronic translator service, further history revealed that the patient recently immigrated as an exchange student from Goyang, South Korea, to West Virginia (WV), arriving in the United States via Washington, D.C., approximately one month before the hospital presentation. He had been living with his host family in WV since his arrival, attending public school. The host household had five snakes of various species, axolotls, cats, dogs, a bearded dragon, and two rats, all of which the patient had direct or indirect contact. He reported being up-to-date with all his age-appropriate vaccinations other than hepatitis vaccines. He had received both the SARS-CoV-2 vaccine and a malaria vaccine almost a full year prior to his current travel to the United States. He denied any history of a known malaria infection. He denied alcohol, tobacco, or drug use. He had a female partner but no reported sexual history.

Initial history did not reveal notable travel within or outside South Korea, but subsequent investigation demonstrated he, along with some family and friends, had traveled to Paju, South Korea, located in Gyeonggi Province approximately three months before arriving in the United States. The group went camping in a rural setting around some stagnant water, and he recalled having many insect bites. Following that trip, the patient did have a short illness, including fever and nausea, which self-resolved. He did not remember anyone else becoming ill during or after that trip.

**Figure 2:**
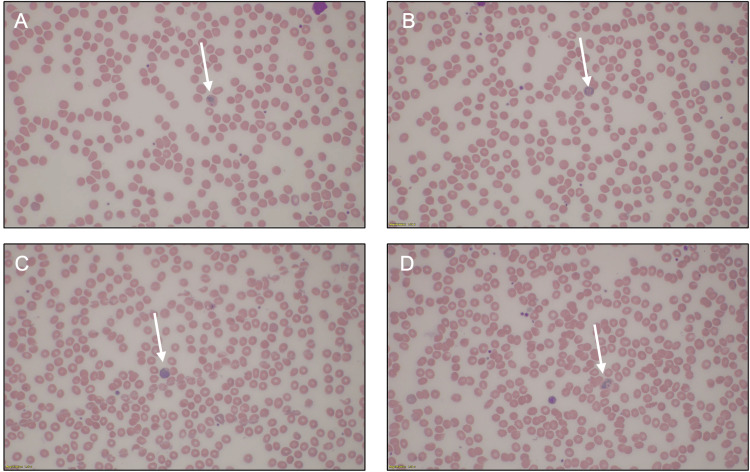
Peripheral smear demonstrating malaria Four representative images of a peripheral blood smear obtained from our patient demonstrating the presence of a malarial parasite, indicated by the white arrow.

Based upon this history and clinical presentation, further infectious workup was recommended by the Peds ID team. Interferon-gamma release assay testing (Quantiferon gold) was negative (with a good mitogen response), a malaria antigen enzyme-linked immunoassay (EIA) was negative, stool culture was without growth, and stool ova and parasites (O&P) were also unrevealing. Thick and thin blood smears were obtained but had to be sent to an outside laboratory for analysis, which delayed the result. An in-facility peripheral blood smear was requested, which revealed normocytic normochromic anemia with slight polychromasia, moderate thrombocytopenia, and the presence of *P. vivax* trophozoite and gametocyte forms with an estimated parasite load (percentage of infected RBCs based on 1,000 RBCs count) of <1% (Figure 2). Ultimately, send-out thick and thin smears were consistent with peripheral smear findings.

Another diagnostic workup was a non-revealing, prompting cessation of ceftriaxone and doxycycline. Upon consultation with the CDC “Malaria Hotline,” the patient was started on hydroxychloroquine sulfate, initially at 800 mg/dose for dose 1 followed by 400 mg/dose for dosages 2, 3, and 4 given at six, 24, and 48 hours after the initial 800 mg/dose. Glucose-6-phosphate dehydrogenase (G6PD), which was found to be within the appropriate range, was measured prior to the initiation of primaquine. Primaquine was prescribed at 30 mg/dose daily for 14 days. The patient demonstrated remarkable progress during his five-day hospital admission with resolved fevers, myalgias, abdominal pain, and resolving jaundice, as well as steadily improving anemia, thrombocytopenia, inflammatory markers, transaminitis, and hyperbilirubinemia. Close outpatient follow-up was coordinated between the PCP and Peds ID team. A follow-up peripheral blood smear seven days into antimalarial therapy was negative for any parasites. Other outpatient labs normalized soon after the completion of primaquine. Follow-up abdominal ultrasound imaging noted resolved hepatosplenomegaly approximately 1.5 months after diagnosis.

Approximately seven months later, the patient returned to our hospital with a fever every other day, vomiting, myalgia, mild scleral icterus, and jaundice. Upon arrival, the patient was febrile with a temperature of 100.7 F, tachycardic up to 120 bpm, and tachypneic with a respiratory rate of 28. On physical examination, the patient was noted to have jaundice and hepatomegaly. Labs obtained demonstrated normocytic anemia with an Hgb of 11.2 and thrombocytopenia with a platelet count of 67,000, which continued to drop to a nadir of 52,000. The patient’s creatine was noted to be elevated at 1.27 mg/dL (baseline, ~0.9 mg/dL). All infectious workups, including blood cultures, were negative. Rapid malaria testing was performed and found to be positive. Thick and thin smears were sent to an outside laboratory. Treatment for malaria began with a one-time dose of 800 mg of hydroxychloroquine followed by a 400 mg dose at intervals of six, 24, and 48 hours after the initial dose. The patient was also treated with a one-time dose of 300 mg tafenoquine under the direction of the institutional Peds ID team and CDC “Malaria Hotline.” Thick and thin smear results ultimately demonstrated a non-*P. falciparum* malaria suspected to be *P. vivax* with a parasitemia of 0.3%. The patient tolerated antiparasitic treatment without issue and was discharged within 72 hours of admission.

## Discussion

According to the World Health Organization (WHO), 269 million cases of malaria occurred worldwide in 2022, 420 of those cases being in South Korea, indicating the rarity of acquiring malaria from this country [5]. In this case report, we described a pediatric case of malaria in a South Korean traveler in rural Appalachia. *P. vivax* can relapse months after the initial illness. This is due to the dormancy of liver hypnozoites, which may reactivate at any time [6]. This was seen in our patient who had a limited one-week illness post-travel to the area of the outbreak in the Gyeonggi Provenance prior to U.S. arrival.

While this patient was vaccinated against malaria, vaccines target *P. falciparum*, not *P. vivax*. This patient also had a significant travel history, which included a recreational excursion to an area later linked with a malaria outbreak. According to the CDC, in South Korea, malaria is rare and normally limited to the rural areas of Incheon, Kangwon-do, and Kyonggi-do provinces [2]. *P. vivax* species is solely responsible for malaria in the known provinces of South Korea. Interestingly, our patient had camped in an area (Gyeonggi Provenance) that had experienced a recent malarial outbreak (260 cases) [2]. Our patient reported a brief, one-week illness after camping that subsequently resolved. This initial illness is seemingly the onset of his parasitic infection. Malaria initially mimics a “viral illness” with symptoms of fever and malaise that typically resolve, with more serious symptoms presenting later [6].

Upon admission, this patient had a negative rapid malaria test. Sensitivity for rapid malarial tests is about 95% but only in parasitemia greater than 100 parasites/uL [5]. This patient’s low parasite burden likely made this diagnostic test show a false negative [7].

Thrombocytopenia is a rare symptom of malaria and is typically mild (between 100,000 and 140,000) when present [3]. However, our patient demonstrated moderate thrombocytopenia. In addition, thrombocytopenia is normally associated with high parasite loads, unlike in this patient, who demonstrated a parasite load of <1% [8]. Despite this, the patient was without other more classic findings of severe malaria, such as impaired consciousness/coma, acute kidney injury, circulatory collapse, and disseminated intravascular coagulation, which would prompt the use of intravenous antimalarial drugs [9, 10].

According to the CDC, patients infected with *P. vivax* or *P. ovale* may experience a relapse of infection months, even years, after initial symptom onset and despite guideline-directed antiparasitic therapy [2]. Despite a low parasite load, guideline-concordant treatment that was well tolerated, and close interval follow-up, this patient relapsed [5]. In addition, the patient had elements of severe malarial illness due to the presence of acute kidney injury, although parasite load again was <1% [2]. Due to potential concerns of related pharmacokinetic and pharmacodynamic limitations of primaquine, a combination of hydroxychloroquine and tafenoquine was used to manage the relapse [2,9].

In summary, this patient contracted malaria during a recent outbreak in South Korea, which has a relatively low number of malaria cases. In addition, this patient tested negative on his initial rapid malaria test, most likely due to his low parasitemia. Despite this negative test, a peripheral blood smear and subsequently confirmatory thick and thin smears were of vital importance as these demonstrated the presence of *P. vivax*. Even with a low parasite load, this patient developed severe thrombocytopenia and significant clinical symptoms. His low parasitemia also complicated diagnostic practices. This case uniquely demonstrates the thorough history of fever in a traveler and the importance of encompassing a differential supported by detailed testing methodology.

## Conclusions

In this case, we discussed a 17-year-old Korean traveler with severe malaria features despite a low parasite burden, which highlights the paramount importance of regional infectious disease variation. Notable barriers to obtaining key historical elements are often associated with language differences despite interpreter services and patient acuity. Despite these barriers, every effort must be made to obtain a detailed and accurate history. Malaria should be included on the differential despite negative rapid malaria testing in an international traveler with tertian fever with notably unexplained transaminitis and hyperbilirubinemia with otherwise ruled-out infectious workup. Prompt treatment and close follow-up are necessary to prevent long-term consequences.
